# Reduction of Pulmonary Air Leaks with a Combination of Polyglycolic Acid Sheet and Alginate Gel in Rats

**DOI:** 10.1155/2018/3808675

**Published:** 2018-01-02

**Authors:** Mari Matoba, Toshitaka Takagi, Hiroyuki Tsujimoto, Yuki Ozamoto, Jo Ueda, Akeo Hagiwara

**Affiliations:** ^1^Medical Life System, Faculty of Life and Medical Science, Doshisha University, 1-3 Tatara Miyakodani, Kyotanabe, Kyoto 610-0394, Japan; ^2^Fushimi Okamoto Hospital, 9-50 Kyomachi, Hushimi-Ku, Kyoto, Kyoto 612-8083, Japan; ^3^Kusatsu General Hospital, 1660 Yabase-cho, Kusatsu, Shiga 525-8585, Japan; ^4^Kainan Hospital, 396 Minamihonden, Maegasu-cho, Yatomi, Aichi 498-8502, Japan

## Abstract

Postoperative air leaks remain a major cause of morbidity after lung resection. This study evaluated the effect of a combination of polyglycolic acid (PGA) sheet and alginate gel on pulmonary air leaks in rats. Four pulmonary sealing materials were evaluated in lung injury: fibrin glue, combination of PGA sheet and fibrin glue, alginate gel, and combination of PGA sheet and alginate gel. With the airway pressure maintained at 20 cmH_2_O, a 2 mm deep puncture wound was created on the lung surface using a needle. Lowering the airway pressure to 5 cmH_2_O, each sealing material was applied. The lowest airway pressure that broke the seal was measured. The seal-breaking pressure in each experimental group was fibrin, 10.4 ± 6.8 cmH_2_O; PGA + fibrin, 13.5 ± 6.5 cmH_2_O; alginate gel, 10.3 ± 4.9 cmH_2_O; and PGA + alginate, 35.8 ± 11.9 cmH_2_O, respectively. The seal-breaking pressure was significantly greater in the PGA + alginate gel group than in the other groups (*p* < 0.01). There were no significant differences among the other three groups. Alginate gel combined with a PGA sheet is a promising alternative to fibrin glue as a safe and low-cost material for air leak prevention in pulmonary surgery.

## 1. Introduction

Postoperative air leaks remain a major cause of morbidity after lung resection. Pulmonary air leaks in thoracic surgery result in prolonged hospitalization and higher hospital costs [1]. Various strategies have been proposed to manage pulmonary air leaks [2–8]; however, their outcomes have been inconclusive. It is difficult to prevent air leaks by suturing or stapling in cases of severe pulmonary emphysema or at sites near the lung hilum. Moreover, some materials for air leak prevention have limitations relating to safety and cost. Fibrin glue alone or in combination with polyglycolic acid (PGA) have been reported as effective materials for preventing pulmonary air leaks [4, 9]. PGA is biodegradable and functions as a scaffold for tissue regeneration. These materials have been used in thoracotomy or thoracoscopic surgeries and applied on staple lines [7, 10]. PGA sheets alone have been reported to reduce pulmonary air leaks [11, 12], while PGA sheets and fibrin glue in combination showed superior effects to prevent air leaks [13, 14]. However, the effects of conventional materials have been inconclusive thus far [4, 15, 16]. A general concern of fibrin glue is the transmission of blood-borne diseases [17, 18]. Moreover, fibrin glue is expensive. In the present study, the sealing effects of alginic acid, which has high safety and low cost, were investigated as a possible alternative for fibrin glue. Few studies have accurately assessed each method in terms of pressure resistance at the time of sealing [19]. In this study, the seal-breaking pressure of this new material was investigated for use in preventing pulmonary air leaks.

## 2. Materials and Methods

Four pulmonary sealing strategies were evaluated in lung injury: (1) fibrin glue, (2) combination of PGA sheet and fibrin glue, (3) alginate gel, and (4) combination of PGA sheet and alginate gel.

### 2.1. Materials

#### 2.1.1. Fibrin Glue

Fibrin glue (Beriplast P Combi-Set®, CSL Behring Co., PA, USA) is composed of solutions A (fibrinogen and aprotinin) and B (thrombin and calcium chloride). When solutions A and B are mixed for use, the fibrin adhesive mimics physiological fibrin clot formation by the coagulation system.

#### 2.1.2. PGA Sheet

**Figure 1:**
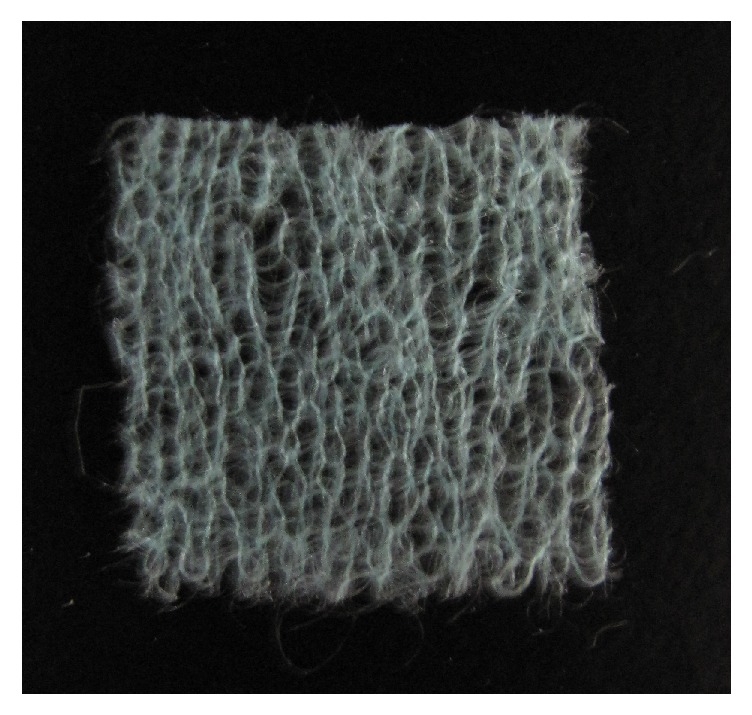
Polyglycolic acid sheet (Neoveil).

A PGA sheet (Neoveil®, Gunze Ltd., Kyoto, Japan) (Figure 1) was cut to 5 mm × 5 mm in size and sterilized with ethylene oxide for 22 hours. The fiber diameter of the PGA sheet (Neoveil) is 16.1 *μ*m, the average distance between fibers is 27.4 *μ*m, and the average sheet thickness is 0.15 mm. Ethylene oxide gas was removed under conditions of decompression for 1 week.

#### 2.1.3. Alginate Gel

Sodium alginate powder (Alto®, Kaigen Ltd., Osaka, Japan) (molecular weight: 32,000–250,000) was dissolved in saline to prepare 5 w/v% alginate solution. The gelling agent consisted of calcium gluconate solution (Calcicol®, Nichi-Iko Ltd., Toyama, Japan). Sodium alginate solution was partially cross-linked with calcium gluconate and gelled, and the alginate gel acquired high local retentivity.

The application of fibrin and alginate alone and in combination with a PGA sheet to the lung injury site is described in Sections 2–4.

### 2.2. Animal Protocol and Experimental Design

The animal experiments were approved by the Doshisha University Animal Experimentation Committee. All surgical procedures and anesthesia protocols were conducted in accordance with the Animal Care Guidelines of Doshisha University. During the experimental period, a week of habituation period was set. Nonpregnant 8-week-old female Wistar/ST rats weighing approximately 200 g were used for this study. All the rats were housed separately and maintained under standard specific pathogen-free conditions (a light-dark cycle of 12 : 12 h, temperature of 20.1–23.5°C, and humidity of 37–65%). Standard laboratory rodent chow and water were freely available. Twenty-four rats were randomly assigned to four experimental groups: (1) fibrin (*n* = 6), (2) PGA + fibrin (*n* = 6), (3) alginate gel (*n* = 6), and (4) PGA + alginate gel (*n* = 6).

### 2.3. Surgical Procedure for Lung Injury

**Figure 2:**
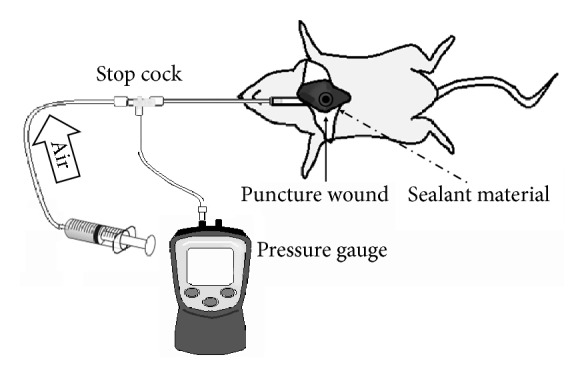
Schema of airway pressure measurement in a rat.

**Figure 3:**
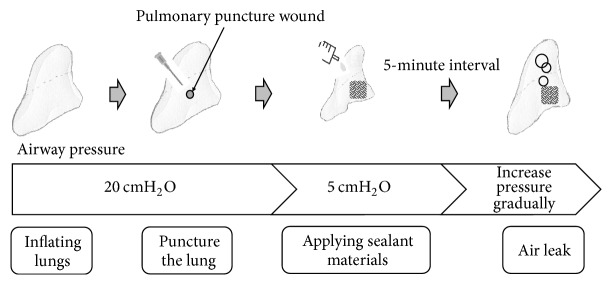
Surgical procedure and pressure measurement.

All rats were killed humanely by intraperitoneal injection of pentobarbital sodium at a fatal dose (0.025 mg/g) under isoflurane inhalational anesthesia. Rats were fixed in the dorsal position. Endotracheal intubation with a 16G catheter was carried out, and the catheter was fixed by ligation. Thoracotomy was performed by removing all the ventral side ribs and incising the diaphragm to expose the lung. The tracheal tube was connected to a pressure gauge (testo510®, Testo SE & Co. KGaA, Lenzkirch, Germany) and syringe with a three-way stopcock (Figure 2). With the airway pressure maintained at 20 cmH_2_O, a 2 mm deep puncture wound was created on the surface of the right middle lung lobe using a 23 G needle. Bleeding was stopped by astriction. After lowering the airway pressure to 5 cmH_2_O, each sealing material was applied (Figure 3).

### 2.4. Application of Sealing Materials (Figure 4)

#### 2.4.1. Fibrin Glue (Fibrin Group)

The pleural puncture wound was sealed by instillation of a drop of fibrinogen solution (solution A), followed by simultaneously spraying fibrinogen and thrombin solutions (solutions A and B, resp.) (total instillation amount of solution A, 0.05 ml; solution B, 0.05 ml).

#### 2.4.2. Combination of a PGA Sheet and Fibrin Glue (PGA + Fibrin Group)

The pleural puncture wound was sealed by instillation of a drop of fibrinogen solution (solution A), and then a PGA sheet was placed onto the wound with a forceps and pressed by an index finger to absorb the solution. Then, fibrinogen and thrombin solutions were simultaneously sprayed over the PGA sheet (total instillation amount of solution A, 0.05 ml; solution B, 0.05 ml).

#### 2.4.3. Alginate Gel (Alginate Group)

The pleural wound was sealed with instillation of 0.025 ml sodium alginate solution followed by five drops of calcium gluconate solution by using a syringe attached to a 26 G needle. The same procedure was repeated twice. After a 5 min interval, another 0.025 ml of sodium alginate solution was instilled.

#### 2.4.4. Combination of PGA Sheet and Alginate Gel (PGA + Alginate Group)

The pleural wound was sealed with instillation of 0.025 ml sodium alginate solution, and then a PGA sheet permeated by a drop of calcium gluconate was overlaid. Four drops of calcium gluconate solution were then instilled onto the PGA sheet, followed by spraying with 0.025 ml sodium alginate solution. After a 5 min interval, 5 drops of calcium gluconate followed by 0.025 ml sodium alginate solution were instilled.

### 2.5. Measurement of Minimum Seal-Breaking Airway Pressure

After application of each sealant, it was left motionless for 5 min, and then the lowest airway pressure that broke the seal (seal-breaking pressure) was measured. Soap water was sprayed over the sealant in advance. Airway pressure was gradually increased at a rate of 2 cmH_2_O/s from 5 cmH_2_O, and the minimum seal-breaking pressure that caused sealing failed was determined by the appearance of a bubble.

### 2.6. Statistical Analysis

Seal-breaking pressure was compared among the groups by using one-way analysis of variance and Tukey's test. Differences were defined to be statistically significant for *p* values less than 0.05.

## 3. Results

**Figure 5:**
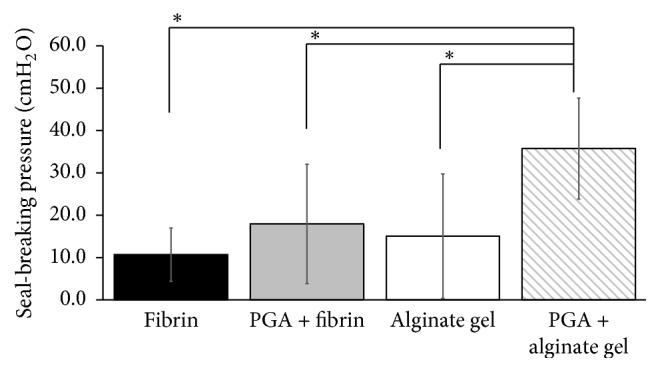
Minimum seal-breaking airway pressure for each sealing material. ^*∗*^ *p* < 0.05.

Seal-breaking pressure in each experimental group (mean ± standard deviation) was as follows: (1) fibrin group, 10.4 ± 6.8 cmH_2_O; (2) PGA + fibrin group, 13.5 ± 6.5 cmH_2_O; (3) alginate group, 10.3 ± 4.9 cmH_2_O; and (4) PGA + alginate group, 35.8 ± 11.9 cmH_2_O. Seal-breaking pressure in the PGA + alginate group was significantly greater than that in the other groups (*p* < 0.01). There were no significant differences among the other three groups (Figure 5).

## 4. Discussion

In this study, the combination of alginic acid gel and PGA sheet showed excellent effects for the prevention of pulmonary air leaks in rats. In recent years, fibrin glue has been widely used for the prevention of air leaks in pulmonary surgery [11, 16]. However, fibrin glue is problematic in that it is derived from human blood and thus is associated with a clinical risk of pathogenic infections. Alginate is a naturally derived polysaccharide typically obtained from brown seaweed and has been safely used for many biomedical applications due to its biocompatibility, low toxicity, relatively low cost, and mild gelation by addition of divalent cations such as Ca2+ [20]. Consequently, alginate is considered a candidate to replace fibrin glue as a sealant against pulmonary air leaks.

The minimum seal-breaking pressures of fibrin glue and alginate gel alone were approximately 10 cmH_2_O in this study, which is insufficient to prevent air leaks during and after pulmonary surgery under mechanical ventilation. By contrast, the substitution of alginic acid gel for fibrin glue in combination with a PGA sheet led to superior air leak prevention. The minimum seal-breaking pressure of the combination of alginate gel and PGA sheet was 35 cmH_2_O, which is thought to provide sufficient strength for air leak prevention under forced ventilation by a respirator.

The combination of PGA sheet and fibrin glue has been reported to provide good air leak prevention [12, 15]. The PGA sheet is flexible and follows the shape of the organs, and it retains liquid, allowing it to be combined with a viscous adhesive to close air leaks. However, in the present study, the combination of PGA sheet and fibrin glue did not demonstrate a significant improvement in air leak sealing effects compared to fibrin glue alone. We speculate that there may be two possible reasons for this discrepancy. First, in a previous study on sealing effects with long-term observation of the combination of PGA sheet and fibrin glue, the PGA sheet functioned as a good scaffold for tissue regeneration to accelerate lung wound healing [14]. The present study did not investigate long-term healing, but only short-term leak prevention, which might have led to the similar results between fibrin alone versus fibrin + PGA. Rapid wound healing is an important factor for air leak prevention after pulmonary surgery, and alginate is known to promote wound healing as a good scaffold for tissue regeneration [21]. Second, in the assessment of direct and instantaneous sealing effects during surgery, it is possible that differences in procedures to apply fibrinogen, thrombin, and PGA sheets affected this discrepancy. It is considered necessary for a glue or gel to fill the fiber gap of a PGA sheet to show a satisfactory air leak prevention effect. However, it is possible that, with the combination of PGA sheet and fibrin glue used in the present experiment, the fibrin glue could not sufficiently fill the fiber gap. Because fibrin glue has poor impregnating ability, solution A was applied to the pleural wound in advance, and then solutions A and B were sprayed simultaneously, as in previous reports. Immediately after mixing the two solutions, the fibrin glue develops high adhesiveness and few bubbles remain within the glue. These physical properties may have inhibited fibrin glue permeation into the PGA fiber gap. The 5% sodium alginate solution has low viscosity and turns into highly adhesive gel crosslinked by calcium [21]. Preinstilled sodium alginate solution in addition to sprayed sodium alginate solution thoroughly permeated the calcium gluconate-dipped PGA sheet. Consequently, the alginate gel filled the fiber gap of the PGA sheet. The sealant was adhered tightly by adding one more layer of alginate gel over the PGA sheet. By applying alginate gel in multiple layers to the lung wound, the sealing effect of the combination of PGA sheet and alginate gel may have been reinforced.

Fibrin glue used alone could easily flow from the application site due to its low retentivity. Sodium alginate solution itself is watery and can be easily handled but is inadequate as a sealant due to poor local retentivity. To increase pressure resistance and local retentivity, sodium alginate was gelated by crosslinking with calcium gluconate as described in the Materials and Methods. As with fibrin glue, the simultaneous use of a PGA sheet allowed the alginate gel to remain at the application site. In this study, the ratio of sodium alginate and calcium gluconate was determined as the concentration that showed superior antiadhesive effects in our previous study [22]. However, the optimal concentration of sodium alginate solution and dosage of calcium gluconate must be evaluated in further investigations.

Sealant-inducing pleural adhesion is a concern in the use of sealant materials to prevent pulmonary air leak. Although pleural adhesions are known to prevent pulmonary air leak, pleural adhesions at rethoracotomies often complicate pulmonary surgeries, making these procedures more time-consuming and hazardous for the patient [23, 24]. The mild acidity of polyglycolic acid, produced during the nonenzymatic degradation of PGA, causes chronic inflammation, and adhesions subsequently occur around the site where the PGA mesh was placed [14, 24]. Although PGA-induced adhesions have long been considered problematic, PGA continues to be used due to its effectiveness as a biomaterial. Therefore, when assessing sealants for air leak prevention, in addition to their sealing effect, antiadhesive effects are also desirable. Fibrin glue is often used to prevent adhesion; however, its effectiveness for this purpose is controversial [22, 25, 26]. We previously reported a superior antiadhesive effect of alginate gel in comparison to fibrin glue in a study of PGA-induced adhesions [22].

The limitations of this study include the fact that it compared the mechanical sealing effect during surgery and not include long-term observation after surgery and that the healing process of the peritoneal injury was not taken into account due to the short-term nature of the intraoperative observation. Furthermore, this study was conducted using an experimental model in rats, and further investigation in humans is necessary to confirm the results.

## 5. Conclusions

In conclusion, alginate gel, a safe and low-cost material, is a potential substitute for the use of fibrin glue in combination with a PGA sheet for air leak prevention in pulmonary surgery. The combination of PGA sheet and alginate gel may be useful to close air leaks at sites of pleural injury near the hilar region, where it is difficult to close air leaks by suturing or stapling, due to their ease of handling during application.

## Figures and Tables

**Figure 4:**
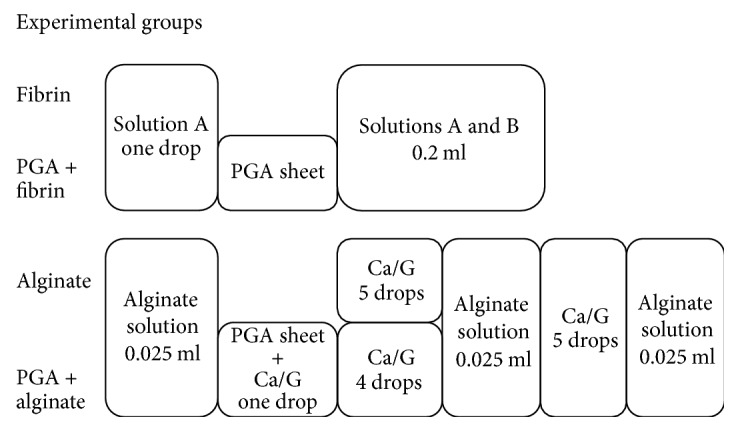
Sealant material application procedures in each experimental group. Ca/G, calcium gluconate.
